# The Molecular Basis of the Sodium Dodecyl Sulfate Effect on Human Ubiquitin Structure: A Molecular Dynamics Simulation Study

**DOI:** 10.1038/s41598-018-20669-7

**Published:** 2018-02-01

**Authors:** Majid Jafari, Faramarz Mehrnejad, Fereshteh Rahimi, S. Mohsen Asghari

**Affiliations:** 1 Nanobiotechnology Lab, Department of Life Sciences Engineering, Faculty of New Sciences and Technologies, University of Tehran, 14395-1561 Tehran, Iran; 2 Department of Biology, Faculty of Sciences, University of Guilan, 4193833697 Rasht, Iran

## Abstract

To investigate the molecular interactions of sodium dodecyl sulfate (SDS) with human ubiquitin and its unfolding mechanisms, a comparative study was conducted on the interactions of the protein in the presence and absence of SDS at different temperatures using six independent 500 ns atomistic molecular dynamics (MD) simulations. Moreover, the effects of partial atomic charges on SDS aggregation and micellar structures were investigated at high SDS concentrations. The results demonstrated that human ubiquitin retains its native-like structure in the presence of SDS and pure water at 300 K, while the conformation adopts an unfolded state at a high temperature. In addition, it was found that both SDS self-assembly and the conformation of the resulting protein may have a significant effect of reducing the partial atomic charges. The simulations at 370 K provided evidence that the SDS molecules disrupted the first hydration shell and expanded the hydrophobic core of ubiquitin, resulting in complete protein unfolding. According to these results, SDS and temperature are both required to induce a completely unfolded state under ambient conditions. We believe that these findings could be useful in protein folding/unfolding studies and structural biology.

## Introduction

Surfactants or surface active agents are used in a broad range of applications in pharmaceutical industries and biological studies, including solubilizing membrane proteins, and in crystallography studies^1–3^. They are extensively used in biochemistry and biotechnology (e.g., in sodium dodecyl sulfate-polyacrylamide gel electrophoresis (SDS-PAGE) and in probing the protein’s folding/unfolding mechanisms)^4–11^. Anionic surfactants are the most commonly used surfactant. Two examples of their applications are denaturing globular proteins and modifying the activities of enzymes. The surfactant-protein interactions depend on the intrinsic properties of both the surfactant and the protein, especially the alkyl chain length and the hydrophilic head group charge of the surfactant^4,8^. SDS is a well-known ionic detergent, consisting of a hydrophobic 12-carbon chain and a polar sulfate head group whose chemical properties and applications are more extensively investigated than the other surfactants^12^. SDS strongly binds to the positively charged and the hydrophobic residues of proteins through its sulfate groups and alkyl chains, respectively^13^. Accordingly, it is a valuable detergent in the field of structural biology and protein folding/unfolding studies. For example, it can induce the formation of α-helices in a protein structure^7,14,15^. SDS induces cooperative unfolding in proteins such as bovine serum albumin (BSA), hen egg white lysozyme, α-lactalbumin, and β-lactoglobulin structures at high concentrations^16–21^. Although SDS can denature proteins, the addition of nonionic surfactants, including octaethylene glycolmonododecyl ether (C12E8) and n-dodecyl-β-D-maltopyranoside (DDM) in the SDS-protein solution, induces refolding of proteins^11^. The nonionic surfactants tend to interact with each other rather than with proteins, and they weakly react with proteins^5^. Additionally, they can protect the conformations of proteins from unfolding induced by the anionic surfactants^5,22^.

The structure of the SDS micelles, which is dependent on the detergent concentrations, can affect the unfolding rate of proteins. By increasing the SDS concentrations, the micellar structure shifts from spherical to cylindrical. Hence, the unfolding rate of mixed α/β proteins in the presence of cylindrical micelles directly depends on the micellar concentration^4^. The structure of the SDS surfactant is similar to the phospholipid molecules of the membrane (i.e., it has a hydrophilic head group and a hydrophobic tail). Therefore, the molecule can mimic a biological membrane environment^23–28^. Moreover, the interactions between proteins and phospholipids are associated with different protein aggregation diseases through promoting or avoiding the aggregations^29–32^.

Ubiquitin is a small and a heat stable protein with 76 amino acid residues, and it contains 11 acidic and 11 basic amino acids along with a mixed α/β secondary structure^29,33^. The protein has been called ubiquitin because it is ubiquitous and can be present on the surface of membranes, in the cytoplasm, and in the nucleus of different eukaryotic cells^33^. Ubiquitin is classified under a family of protein modifiers, which have the same structure but different amino acid sequences^34–36^. In different organisms, the amino acid sequence of ubiquitin varies by only a few residues; therefore, it is considered as one of the highly conserved proteins in eukaryotic organisms^33,37^. In eukaryotic cells, ubiquitin is involved in proteolytic and non-proteolytic pathways. In the proteolytic pathway, the protein gets covalently attached to a target protein which leads to the transportation of the protein to the proteasome system^34,37^. In the non-proteolytic pathway, however, ubiquitin is engaged in activities such as membrane protein transport^38^, DNA repair^39^, removable of abnormal proteins by chaperones^40^, and protein folding^41^, among many other crucial activities^37,42,43^.

The aim of the present study was to investigate the responses, the conformational changes, and the unfolding mechanisms of human ubiquitin in the presence of SDS surfactant at the atomic level of detail. To better understand the effects of SDS on ubiquitin structure, we additionally conducted two independent all-atom molecular dynamics (MD) simulations of ubiquitin in pure water at two ambient temperatures. The results indicated that in the presence of high SDS concentrations and high temperature, human ubiquitin completely lost its secondary structures and adopted a random coil structure, while the SDS molecules stabilized the protein’s native structure at low temperature. The results also revealed that at high temperatures the SDS surfactants adopt a membrane-like structure and the hydrophobic core of the protein is destroyed by SDS molecules.

## Computational Methods

### Molecular dynamics simulation

**Figure 1:**
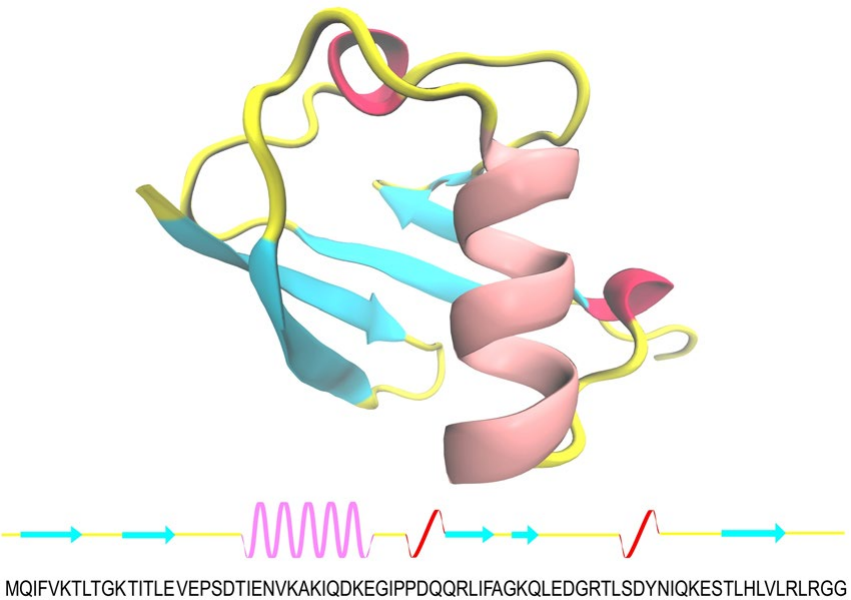
(Upper) the native structure of ubiquitin as a new cartoon model. (Lower) a representative the sequence chain view of ubiquitin; each secondary structure is shown as following color schemes: beta sheets (cyan), alpha helix (pink), 3/10-helix (red), and turn, beta bridge, and bend (yellow).

All MD simulations were conducted using the GROMACS 4.5.6 simulation package^44–46^. The initial coordinates of the human ubiquitin were taken from the Protein Data Bank (PDB ID: 1UBQ) (Fig. 1)^33^. The primary structure of the SDS molecule was obtained from the previous study^47^, and its partial atomic charges and parameters were obtained from the automated force field topology builder database (ATB)^48^, which is based on the GROMOS96 53A6 force field. For S5 and S6 simulations, we first generated the itp file of SDS by the ATB server then reduced the partial atomic charges of each SDS atom based on the partial atomic charges created by the PRODRG server^49^.

The GROMOS96 53A6 force field was applied to all simulations and the simple point charge (SPC) water model was used to provide the solvation conditions for ubiquitin^50^. All systems were electrostatically neutralized using the counter-ions Cl^-^ and Na^+^. The periodic boundary conditions were considered to avoid possible boundary effect problems. The Particle Mesh Ewald (PME) algorithm and a 1.2 nm distance cutoff were applied for the long-range and the short-range electrostatic interactions^51^, respectively. The temperature and pressure were retained using a Nose–Hoover algorithm (at 300 K and 370 K) and a semi-isotropic Parrinello–Rahman algorithm (at 1 atm)^52–54^, respectively. In all MD simulation systems, the steepest descent method was considered for energy minimization and each system was equilibrated under NVT-ensemble and NPT-ensemble states. Further information for all MD simulations is listed in Table 1.

**Table 1:** Molecular dynamics simulations with further details^1^.

System	Temperature	Acronym	Length (ns)	Box size (A^0^)	no. of waters	no. of ions	no. of SDS	SDS concentration (mol/lit)
UBQ^2^ in Water	300	S1	500	52*52*52	4337	10 Na^+^, 10 Cl^−^	—	—
UBQ in Water	370	S2	500	52*52*52	4337	10 Na^+^, 10 Cl^−^	—	—
UBQ in SDS	300	S3	500	52*52*52	2623	121 Na^+^, 10 Cl^−^	111	≈1.3
UBQ in SDS	370	S4	500	52*52*52	2623	121 Na^+^, 10 Cl^−^	111	≈1.3
UBQ in SDS^3^	300	S5	500	52*52*52	2623	121 Na^+^, 10 Cl^−^	111	≈1.3
UBQ in SDS^3^	370	S6	500	52*52*52	2623	121 Na^+^, 10 Cl^−^	111	≈1.3

^1^One of the replication for S1, S2, S3, and S4 systems was extended for 1000 ns, and each simulation was done three times.

^2^UBQ is human ubiquitin.

^3^S5 and S6 simulations with reducing the atomic partial charges, respectively.

### Calculation of free energy

The g_mmpbsa tools were used to calculate the binding free energy, which calculates the free energy using the Molecular Mechanics-Poisson Boltzmann Surface Area (MM-PBSA) method^55,56^. In principle, the binding free energy of each system can be calculated using the following equation (Eq. 1)

1 $${\rm{\Delta }}{G}_{binding}={G}_{complex}-{G}_{ubiquitin}-{G}_{SDS},$$

where Δ*G*_*binding*_ is the total free energy of the complex, minus the total free energy of each complex component in its free state. This value is equal to the total polar binding free energy plus the total non-polar binding free energy of the complex. Therefore, the following equation was used to calculate the average binding free energy (Eq. 2)

2 $${\rm{\Delta }}{G}_{binding}={\rm{\Delta }}{G}_{pb}+{\rm{\Delta }}{G}_{npb}$$

where ΔG_pb_ and ΔG_npb_ are the polar binding free energy and the non-polar binding free energy, respectively, and can be obtained as (Eqs 3, 4)

3 $${\rm{\Delta }}{G}_{pb}={\rm{\Delta }}{G}_{pols}+{\rm{\Delta }}{G}_{elec},$$

4 $${\rm{\Delta }}{G}_{npb}={\rm{\Delta }}{G}_{vdw}+{\rm{\Delta }}{G}_{npols}$$

where ΔG_pols_ and ΔG_npols_ are polar and non-polar energies of solvation, respectively. ΔG_elec_ is the electrostatic energy and ΔG_vdw_ is the van der Waals energy. In the MM-PBSA method, a specified number of snapshots of trajectory, based on the number of steps in the trajectory, is used to calculate the average binding energy of the molecular dynamics system^57^. In each system, 313 snapshots during the simulation time were extracted for the calculation of the average binding energy because the number of steps in the molecular dynamics trajectory was 313 steps.

## Results and Discussion

### Effects of SDS molecules on the secondary structures, compactness, and tertiary structure of ubiquitin

**Figure 2:**
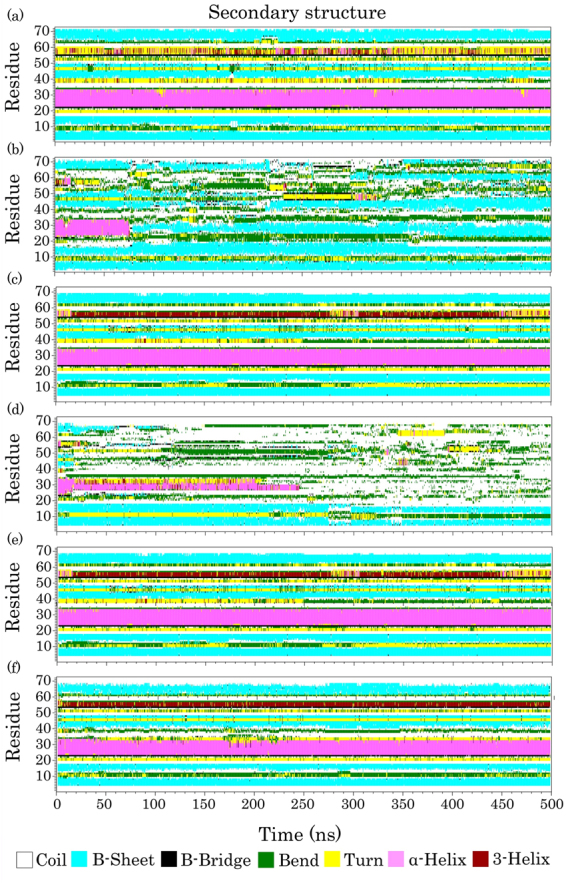
The time evolution of the secondary structure of ubiquitin in (**a**) ubiquitin in pure water at 300 K (S1), (**b**) ubiquitin in pure water at 370 K (S2), (**c**) ubiquitin in aqueous SDS solution at 300 K (S3), and (**d**) ubiquitin in aqueous SDS solution at 370 K (S4), (**e**) ubiquitin in aqueous SDS solution at 300 K (reduced partial atomic charges, S5), (**f**) ubiquitin in aqueous SDS solution at 370 K (reduced partial atomic charges, S6).

Ubiquitin is a heat-stable protein, and its thermal stability depends on ambient pH. However, at neutral pH (our simulation conditions) its melting temperature is ≥100 °C^58^. We first performed the Dictionary Secondary Structure of Proteins (DSSP) analysis to identify the secondary structures of each ubiquitin residue in all MD simulations (Fig. 2). As observed, the ubiquitin secondary structures began to unfold at 370 K and lost its helical contents in pure water, which is in agreement with the aforementioned experimental study^58^. Probably, the fully unfolded state can be observed with an increased simulation time scale, as in one of the replications, it was observed after approximately 300 ns of MD simulation (Supplementary Figure S1). The DSSP analyses also revealed that the secondary structure elements are intact in the pure water at 300 K (Fig. 2(a)).

Above the critical micelle concentration (CMC), SDS can induce or maintain the helical structures of proteins^23,59^. Gregory *et al*. used capillary electrophoresis (CE) and a circular dichroism (CD) spectrophotometer to understand the SDS-ubiquitin interactions. They have also indicated that the increased SDS molecule counts in the ubiquitin-SDS_n_ complex (e.g., ubiquitin-SDS_11_, ubiquitin-SDS_25_, and ubiquitin-SDS_33_) increases the alpha-helical contents of ubiquitin with respect to the native structure^60^. Moreover, in the SDS concentrations below 2 mM, in which ubiquitin bonds to approximately 11 SDS molecules, the protein maintains its native-like secondary structures. As shown in Fig. 2(c), the helical structure of ubiquitin in the presence of SDS was maintained at 300 K, and the protein retained its native-like structure in the presence of 111 SDS molecules ([SDS] ≈ 1.3 M, the CMC value for SDS in pure water is 7–8 mM) during the 500 ns MD simulation. To confirm this result, we extended one of the simulation replications to 1000 ns so that the native structure of the protein was still maintained (Supplementary Figure S2). Previous studies have revealed that the β-sheet strands of proteins are stable against SDS surfactants^5,61^. In good agreement with these results, Fig. 2(c) shows that in the presence of SDS the β-sheet strands of ubiquitin were maintained during the MD simulations at 300 K. An experimental study on human serum albumin (HSA) has also indicated that beyond 90 °C (~360 K) the helical structures of the protein were disrupted in the presence of SDS^9^. However, at temperatures below 80 °C (~353 K), the helical structure of the protein could be more effectively protected at a low concentration of SDS (1:20 molar ratio of SDS to HSA) than in the absence of SDS. As shown in Fig. 2, ubiquitin began to unfold gradually in the SDS micelles at 370 K. Hence, with increasing the simulation time, the protein completely lost its α-helix and most of its β-sheet contents and adopted a random coil structure (Fig. 2 and Supplementary Figure S1).

**Figure 3:**
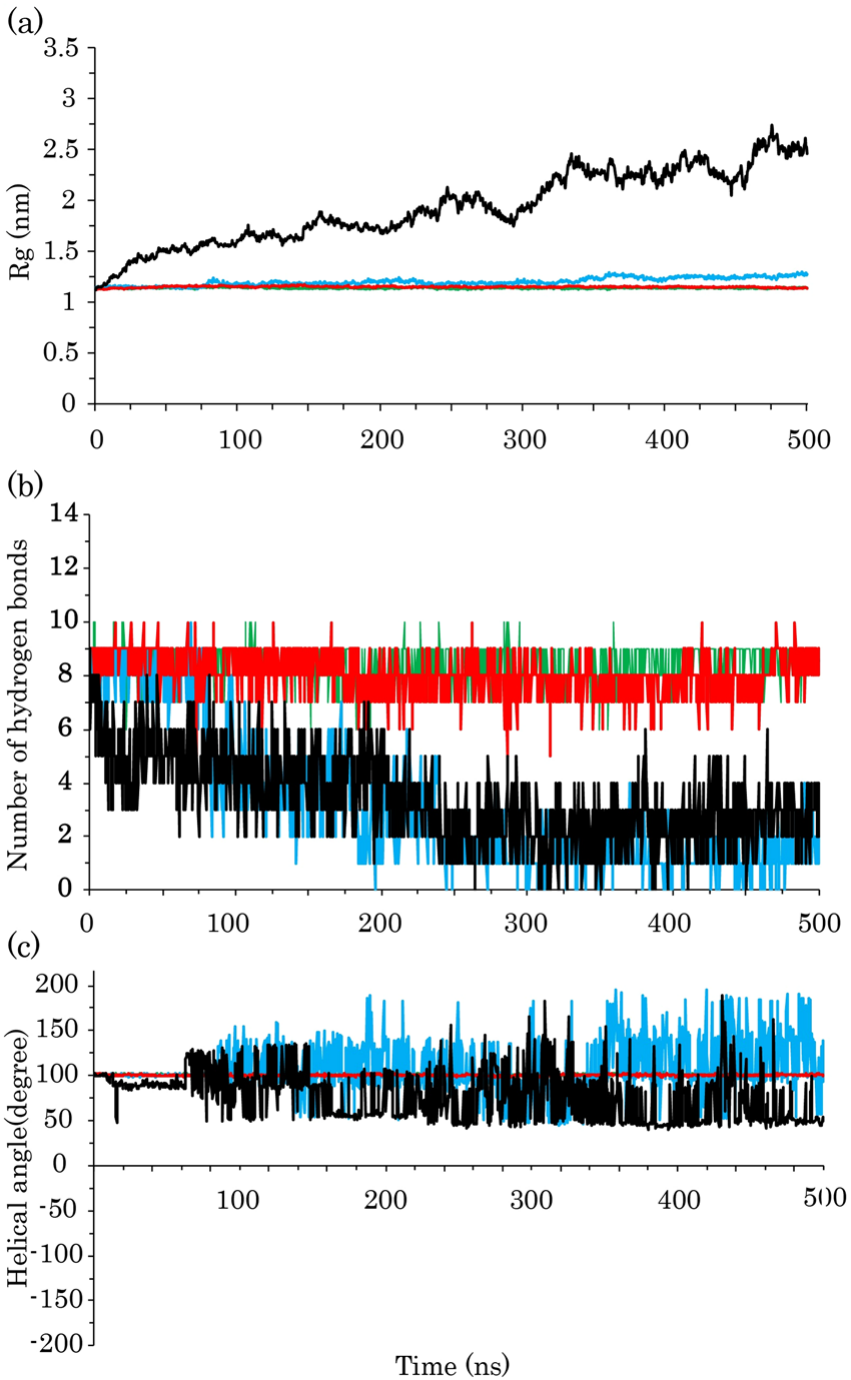
(**a**) The average of all replications for the radius of gyration of ubiquitin, (**b**) The median number of all replications for the internal hydrogen bonds of ubiquitin helix, and (**c**) The average of helical angle per ubiquitin residues. Ubiquitin in water at 300 K, ubiquitin in water at 370 K, ubiquitin in SDS at 300 K, and ubiquitin in SDS at 370 K are shown as green, blue, red, and black lines, respectively.

The radius of gyration (Rg) analysis can provide information about the structural compactness of proteins. Therefore, to identify the compactness changes of ubiquitin, we conducted the Rg analysis for all simulations. As observed in Fig. 3(a), in S2 and at most points over the simulation, the Rg values of ubiquitin were larger than those of the S1 and S3 simulations. The Rg values of the protein in the S1 and S3 simulations were nearly constant, while these values in the S4 simulation were remarkably larger than the others and steadily increased during the MD simulations. These larger values in the S4 simulation were expected because the protein adopted a random coil structure, considering the results of the DSSP analysis (Fig. 2(d) and Supplementary Figure S1). Moreover, the average of Rg and solvent accessible surface areas (SASA) were calculated for all simulation replications (Table 2). As shown in Table 2, the ubiquitin conformation in the presence and absence of the SDS molecules at 300 K has the lowest SASA and Rg values, and this suggests a high compactness in the protein structure when compared to 370 K. As mentioned previously, at high temperatures and SDS concentrations, the responses of the protein to the surfactant completely changed and it lost the α-helix and β-sheet structures under ambient conditions. Consequently, during the 500 ns simulation time, the high temperature cannot be the only factor for denaturing the protein structure because it has the β-sheet strands in pure water at 370 K (Fig. 2(b)). In addition, both SDS and high temperature caused global unfolding and the protein could not be stable under these environmental conditions, even for the β-sheet secondary structures that are usually SDS resistant. It seems SDS acts as a cooperative factor for the thermal denaturation of the protein. This is in agreement with the experimental studies^9^.

**Table 2:** Represents the average values of SASA and Rg in all simulations.

System	Temperature	Rg value (nm)	STD^1^	SASA value (nm^2^)	STD
UBQ in Water	300	1.14	±0.007	47.5	±0.81
UBQ in Water	370	1.21	±0.04	52.3	±1.94
UBQ in SDS	300	1.15	±0.008	48.3	±1.01
UBQ in SDS	370	1.93	±0.36	77	±9.04

^1^Standard deviation (nm).

The helical structures are retained by the intramolecular hydrogen bonding network that is independent of the tertiary structure of proteins. Therefore, they can be effectively disrupted by the SDS surfactants. The rate of denaturation of the β-sheet strands depends on the total unfolding of the proteins because they can make global contacts in the protein structure^5^. To determine the stability of the helical structures in the simulations, we calculated the number of internal hydrogen bonds in the ubiquitin helices and as the average of helical angle per residue. As demonstrated in Fig. 3(b,c), the number of hydrogen bonds in both S2 and S4 simulations was extensively reduced, implying the decrease in the α-helical content under these conditions. However, the value in the S1 simulation was nearly the same as the S3 simulation and the median number of hydrogen bonds for the helical segment was eight. For an α-helix structure, the value of the helical angle is 100°. It is clear that at 300 K the helical angle values were nearly constant and had an average of approximately 101° in all replications (Fig. 3(c)). In the S2 and S4 simulations, a wider distribution of helical angles can be observed, suggesting that there are unstable helical structures.

**Figure 4:**
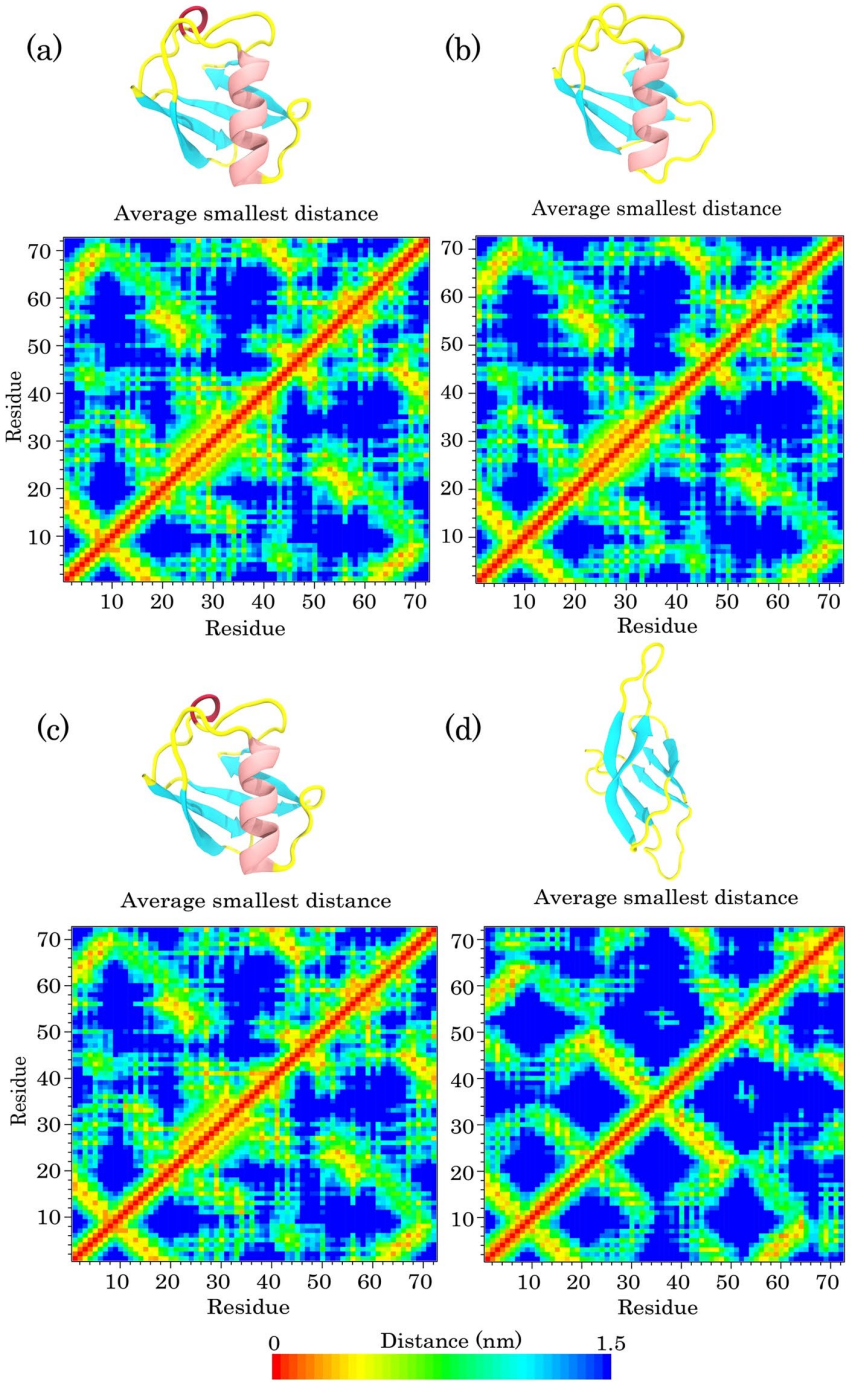
The average minimum distances between residue pairs of ubiquitin for: (**a**) first and (**b**) last snapshot of the protein in water at 300 K; (**c**) first and (**d**) last snapshots of the protein in water at 370 K. Snapshots of ubiquitin extracted from the related steps of trajectory are shown as a cartoon model at the top of each distance matrix.

**Figure 5:**
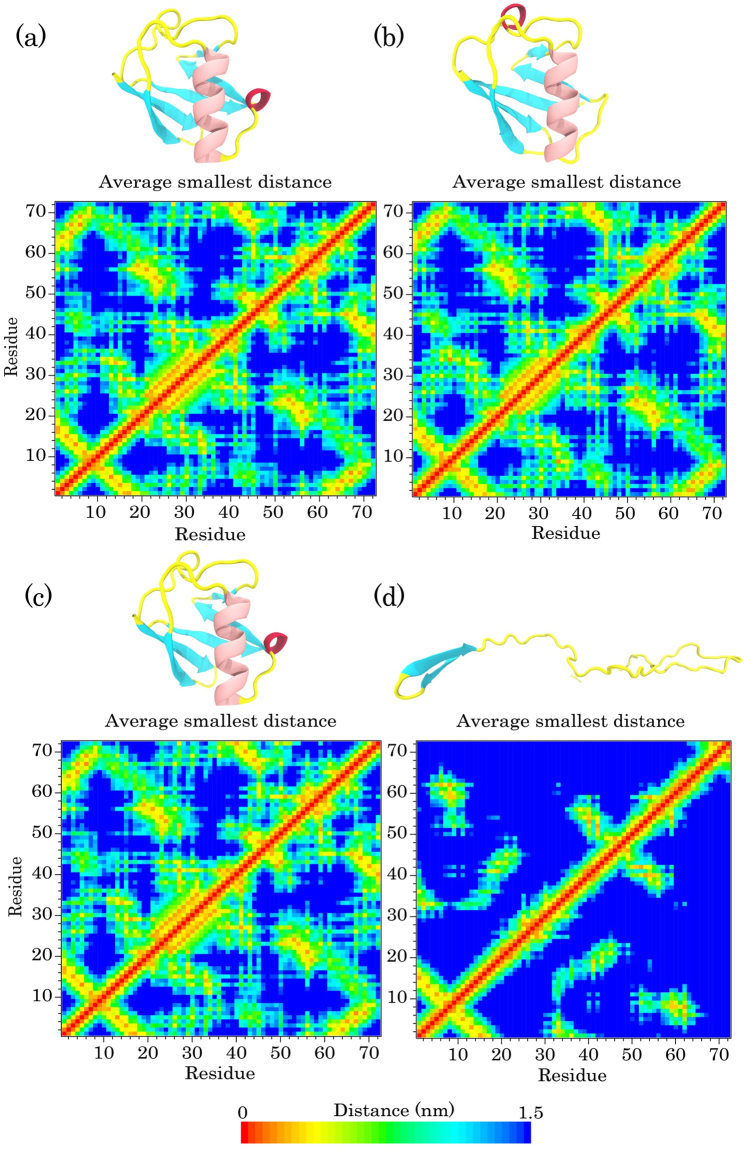
The average smallest distances between amino acid pairs of ubiquitin for: (**a**) first and (**b**) last snapshot of the protein in SDS at 300 K; (**c**) first and (**d**) last snapshots of the protein in SDS at 370 K. Snapshots of the protein extracted from the related steps of trajectory are shown as a cartoon model at the top of each distance matrix.

To identify the tertiary structure changes in all simulations, we computed the average smallest distances between the residue pairs of ubiquitin in the first and last snapshots of all simulations (Figs 4 and 5). As shown in Fig. 4(a,b), the minimum distance matrix of the first snapshot was the same as the last step in the S1 simulation, which suggests that the protein maintained its native conformation under this condition. In the S2 simulation, the blue regions in the distance matrix of the last snapshot were more than that of the first snapshot, which indicates an increase in the smallest distance between the pair of residues (Fig. 4(c,d). The total pattern of the matrix was not changed remarkably, which confirms a partial maintenance of the globular structure and the local unfolding in the protein conformation. We also observed the same behavior as in the S1 simulation for the protein in the S3 simulation (Fig. 5(a,b)). The wider blue area in Fig. 5(b) indicates that there are more distances between residues than the first step and confirms the occurrence of global unfolding in the S4 simulation (Fig. 5(c,d)).

### Free Energy Landscape

To further investigate of the stability and compactness of the ubiquitin structure, we calculated the free energy landscape (FEL), which can be obtained by the following equation (Eq. 5):

5 $$\Delta {{G}}_{{A}}=-{{K}}_{{B}}{T}.ln\frac{{{P}}_{{A}}}{{{P}}_{{B}}},$$

where A is the order parameter, which in the present study is Rg and the C_α_ root mean square deviation (RMSD). P_A_ and P_B_ are the probabilities of finding the system in the A and B states, respectively. P_B_ is the maximal probability. Δ*G*_*A*_ is the corresponding free energy at the A state and K_B_ and T represent the Boltzmann constant and temperature, respectively.

**Figure 6:**
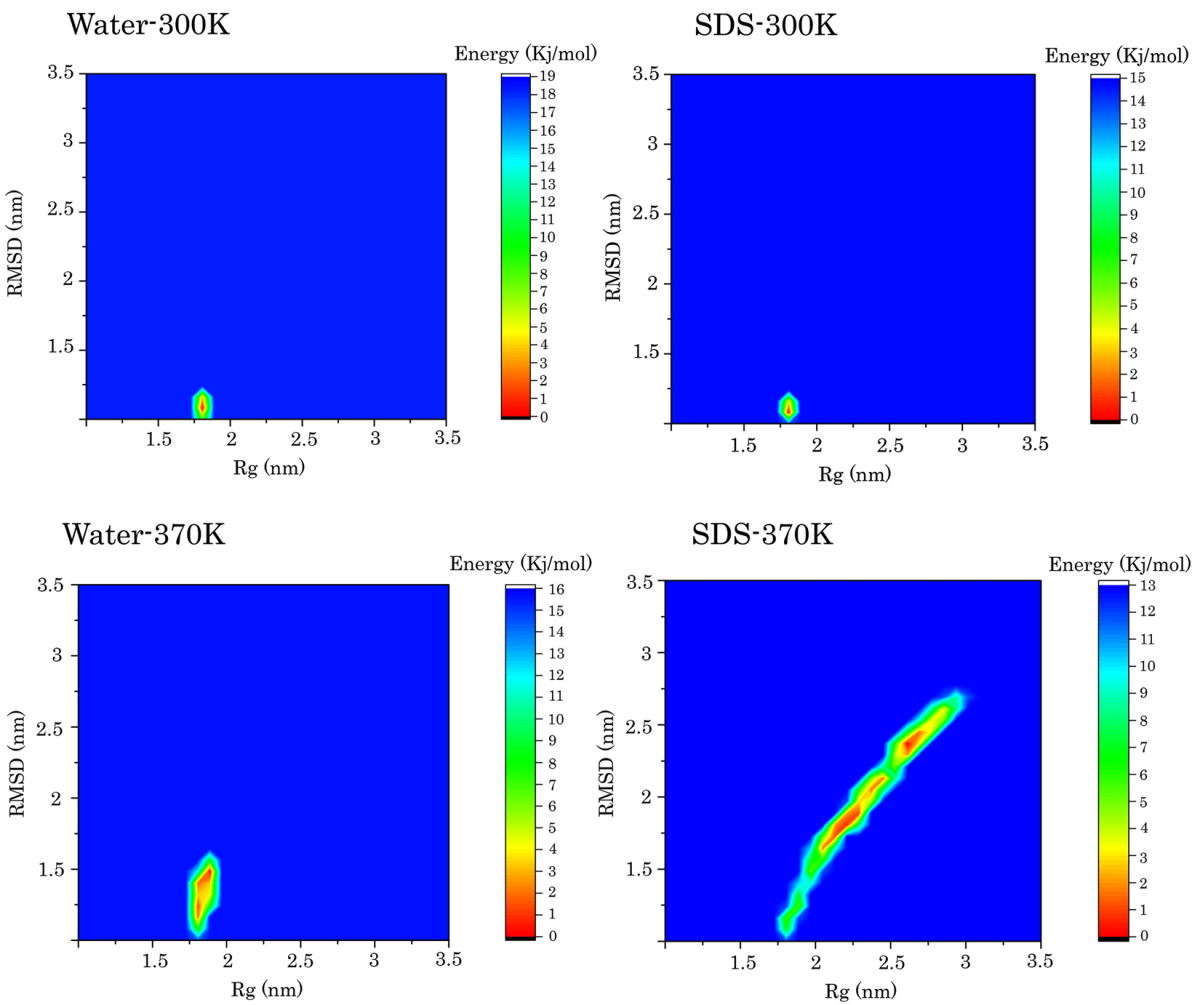
The two-dimensional free energy landscape based on RMSD and Rg of ubiquitin in (**a**) Water 300 K, (**b**) Water 370, (**c**) SDS 300 K, (**d**) SDS 370 K. Please note that the RMSD and Rg values are the average values of all simulation replications. The standard deviation for Rg in (**a**), (**b**), (**c**), and (**d**) was ± 0.007 nm, 0.008 nm, 0.40 nm, and 0.036 nm, respectively. The standard deviation for RMSD in (**a**), (**b**), (**c**), and (**d**) was ± 0.03 nm, 0.020 nm, 0.16 nm, and 0.05 nm, respectively.

SDS has been reported as a stabilizer of the protein structure for STY3178, a mixed α/β protein^10^. The contour plots of FEL in Fig. 6(a,b) shows that the protein was stable at 300 K and the range of green basins was smaller in the presence of SDS compared to its absence. This indicates that in the S3 simulation, the protein structure is slightly more stable, and it has the smallest values for Rg and RMSD compared to those in the simulations. Furthermore, in the S1 and S3 simulations, a deeper valley (ΔG = 0), centered around 1.2 nm and 0.22 nm, was observed. The results suggested that the most stable conformation of human ubiquitin could be obtained when the Rg and RMSD values are approximately 1.2 nm and 0.22 nm, respectively. In the S2 and S4 simulations (Fig. 6(c,d)), the deeper valley did not assign to a single point, which demonstrates the minimum free energy (ΔG = 0) becomes unstable at 370 K.

A wider distribution along the RMSD axis can be observed in the S2 simulation compared to the S1 and S3 simulations. It confirmed the local unfolding of the protein because the compactness of the protein (Rg) did not significantly change when compared with the S1 and S3 simulations. Moreover, there were the higher fluctuations in the protein structure. Finally, in the case of the S4 simulation, the green, yellow, and red basins of the FEL plot extend along the RMSD and Rg axes, which show the highest fluctuations of ubiquitin at high SDS concentrations and high temperatures. Moreover, it confirmed that the protein completely lost its native compactness and, therefore, global unfolding occurred under this condition.

### The effects of micelle structures on the ubiquitin conformation

One of the important issues that affect the unfolding mechanism of proteins by SDS surfactants is the micellar structures. Changes in the structure of micelle can affect the denaturing power of the SDS molecules so that the lower power has been dedicated to simple micelle structures^4^. The binding of a detergent to the protein in the monomeric form may induce some local changes. However, the global unfolding of the protein occurs only in higher detergent concentrations^21^.

**Figure 7:**
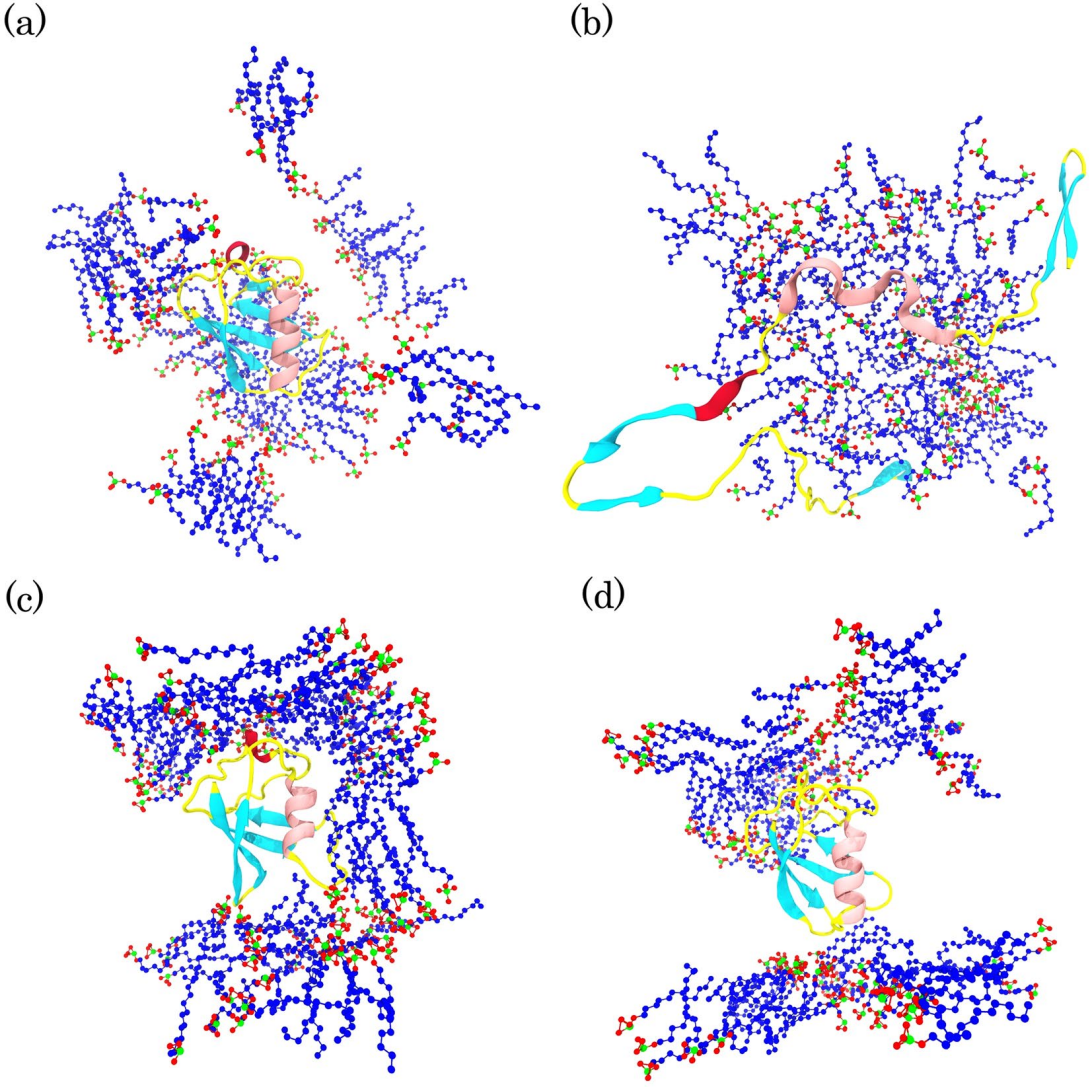
The last snapshots of ubiquitin and SDS micelles in (**a**) ubiquitin in SDS at 300 K, (**b**) ubiquitin in SDS at 370 K, (**c**) ubiquitin in SDS at 300 K; reduced partial atomic charges, and (**d**) ubiquitin in SDS at 370 K; reduced partial atomic charges. The hydrophobic tails of SDS, oxygen atoms of the headgroups, and sulfur atoms are colored in blue, red, and green, respectively. For clarity, water molecules and ions are ignored.

In the present study, the SDS surfactants were used at concentrations above the CMC (111 SDS molecules, [SDS] ≈ 1.3 M). In this condition, SDS molecules are expected to induce denatured states in the protein structure by global contacts. Under these conditions, in the SDS micelles, some independent simple micelle structures have been formed so that all of them could not continuously interact with the protein (Fig. 7(a)). These micellar structures can induce local changes, but they are unable to induce global protein unfolding. In the S5 simulation, the SDS molecules are uniformly distributed around the protein conformation and may induce protective effects on the tertiary structure of the protein (Fig. 7(c)). In this simulation, the hydrophobic tails of SDS molecules saturated the surface of ubiquitin more than in the S3 simulation, which agrees with the results of the non-polar binding energies (Table 3).

**Table 3:** The average binding energy of ubiquitin-SDS complex during the entire simulation time scale^1^.

Energetic components	UBQ-SDS^*^ at 300 K (Kj/mol)		UBQ-SDS at 370 K (Kj/mol)	
SystemFree energy	S3	S5	S4	S6
**ΔG**_**elec**_	16.4	218.5	−10.6	156
**ΔG**_**ps**_	758.3	350	396.4	647.9
**ΔG**_**pb**_	774.7	568.5	385.8	803.9
**ΔG**_**vdW**_	−399.3	−622.7	−720.9	−288.4
**ΔG**_**nps**_	−19.9	−48	−50.3	−14
**ΔG**_**npb**_	−419.2	−670.7	1771.2	−302.4
**ΔG**_**binding**_	355.5	−102.2	−385.4	501.5

^*^Ubiquitin in aqueous SDS solution.

^1^The polar binding energies (**ΔG**_**pb**_), non-polar binding energies (**ΔG**_**npb**_), and total binding energies (**ΔG**_**binding**_) are calculated using (Eq. 3), (Eq. 4), and (Eq. 2), respectively.

**Figure 8:**
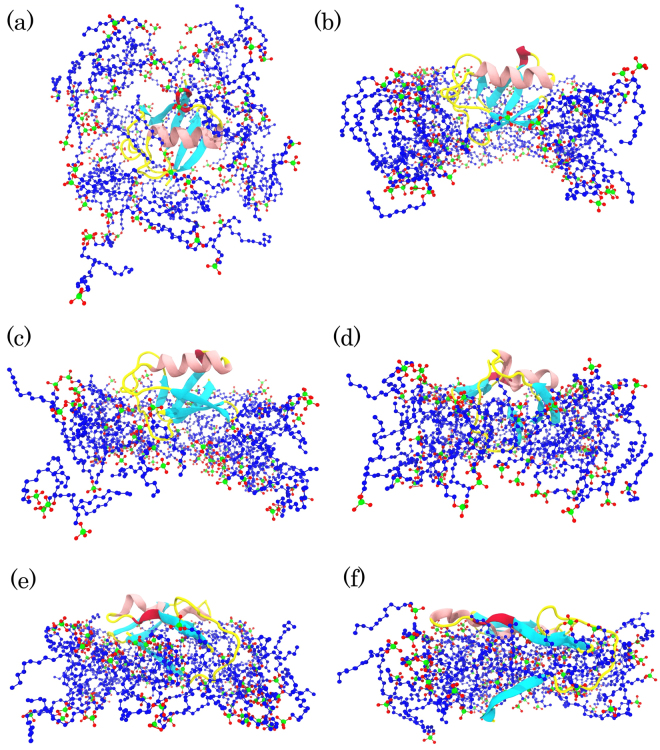
Specified numbers of snapshots extracted from S4 simulation and the simulation time increase from (**a**–**f**). Ubiquitin is shown as a cartoon model and, for clarity, water molecules and ions are not shown.

The structure of micelles in the S6 simulation was different from that in the S4 simulation and could not induce significant changes in the protein conformation (Fig. 7(d)). The SDS surfactants adopted a membrane-like structure at high concentrations and high temperature (S4) and they gradually repelled ubiquitin out of the hydrophobic core. Therefore, the protein was completely unfolded on the hydrophilic surface of the SDS mimic-membrane (Fig. 8(a–f)). The low tendency to keep ubiquitin in the hydrophobic core is reasonable because ubiquitin is a very soluble protein and it needs hydrophilic surfaces to bind. Therefore, it moved to the hydrophilic interface which is composed of the SDS headgroups and water molecules. Previous studies have indicated that the local unfolding of proteins occurs with the monomeric forms of detergent, whereas surfactants lead to global unfolding at concentrations above the CMC^4^ or in the formed micelles^5^. The global unfolding of ubiquitin in the presence of SDS molecules at high temperatures could be because of the global binding of the SDS micelles to the protein, as observed in Fig. 7(b).

The protein denaturing potency of negatively charged surfactants depends on their total negative charges in the micellar form^5^. Therefore, it could be that in the S4 simulation, the global electrostatic interactions with ubiquitin play important roles in the protein unfolding (see Table 3).

### The step by step unfolding of ubiquitin

Previous studies have indicated that the hydrophobic core of ubiquitin is composed of the Ile3, Val5, Ile13, Leu15, Val17, Ile23, Val26, Ile30, Leu43, Leu50, Leu56, and Leu67 residues^62^. They have also found that the ubiquitin core, specifically Val26, is critical for the conformational stability of the protein. It has been shown that binding of the SDS micelles to mixed α/β proteins initially extends the protein structure, leading to the denaturation of protein slightly before the global unfolding^4^. To find the main factor in the ubiquitin unfolding with the SDS micelles at 370 K, we first investigated the roles of electrostatic attractions and then explored the effects of the SDS molecules on the hydrophobic core of the protein. A previous experimental study indicated that the acetylation of lysine residues inhibits the binding of SDS to human ubiquitin. Additionally, it has been shown that the electrostatic attractions are crucial for the binding of SDS molecules to the protein^29^. Anand *et al*. have established that initial electrostatic interactions between the SDS sulfate head groups and the oppositely charged residues of HSA induce partial denaturation in the protein structure^6^. To obtain more insight into the roles of the electrostatic interactions in the unfolding mechanism of ubiquitin, we reduced the partial atomic charges of the SDS molecule and repeated the MD simulations at 300 K and 370 K. Interestingly, our results indicated that the human ubiquitin maintained its native conformation in both simulations. Based on the DSSP analyses (Fig. 2(e,f)), it seems that reducing the partial atomic charges causes SDS to act as a stabilizer for the protein structure at either 300 K or 370 K. These results imply the relevance of the electrostatic interactions in the unfolding mechanism of the protein.

**Figure 9:**
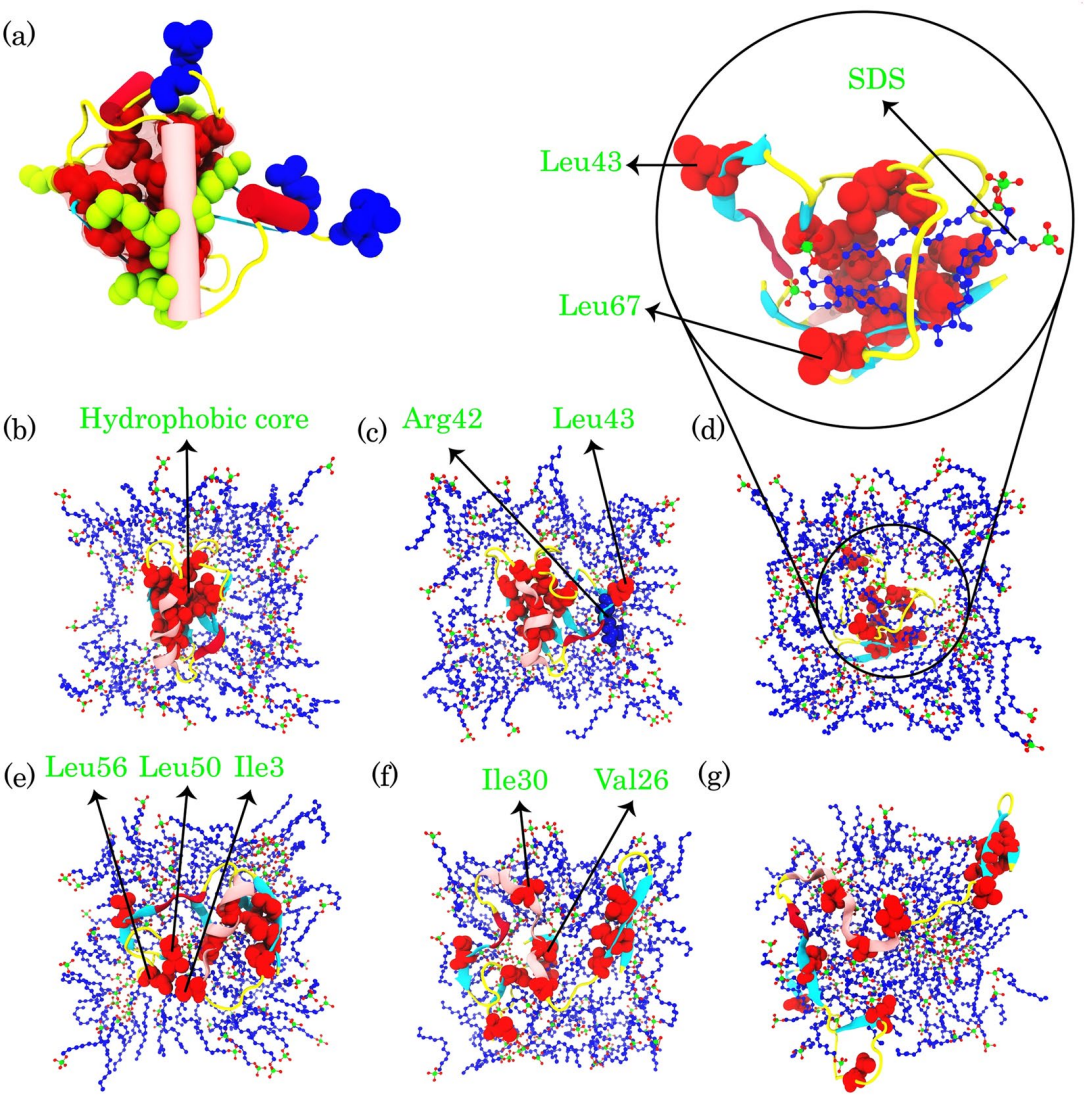
(**a**) The native structure of human ubiquitin and its hydrophobic core (sphere models colored in red), arginine residues (sphere models colored in blue), lysine residues (sphere models colored in yellow). (**b**–**g**) represent all steps of the unfolding mechanism of ubiquitin in SDS at 370 K. Residues in the hydrophobic core are shown as a sphere model and colored in red. (**d**) Only those SDS molecules that inserted in the hydrophobic core are shown in zoom out.

As seen in Fig. 9(a), the basic residues of ubiquitin are distributed throughout the tertiary structure of the protein and are surrounded by the hydrophobic core. In the S4 simulation, the affinity of basic residues to make strong electrostatic interactions with SDS molecules was greater than in the S6 simulation because the SDS molecules had higher partial atomic charges (Table 3). The repulsions between the anionic side chains and the SDS sulfate groups can rule out the unfolding rate and stability of mixed α/β proteins^4^. Therefore, increasing the partial atomic charges in the S4 simulation would also increase the electrostatic repulsions between the acidic residues and the head groups of the SDS molecules. However, increased temperature and hydrophobic interactions may also be involved.

Previous studies have shown that SDS binds to the protein structure in the positively charged surface areas and then alters the binding to the neighboring hydrophobic residues^8,29^. Therefore, the stronger electrostatic interactions in the S4 simulation, as well as a high temperature, could induce the structural stress and may lead to Leu43, which is next to Arg42, leaving the hydrophobic core as the starting point of the unfolding process (Fig. 9(c)). As mentioned, the hydrophobic core of ubiquitin contributes to the protein’s stability. Therefore, it is likely that the ubiquitin hydrophobic core is a good attack point for the SDS molecules at 370 K.

After Leu43, the hydrophobic interactions of the SDS tails affect the internal interactions of Leu67 with the hydrophobic core and result in the removable of the residue from the hydrophobic core (Fig. 9(d)). Leu43 and Leu67 leaving the hydrophobic core provides an opportunity for the SDS molecules to insert into the hydrophobic core of the protein (zoom out in Fig. 9(d)). These molecules affect the internal hydrophobic interactions and lead to Ile23, Leu50, and Leu56 leaving the hydrophobic core (Fig. 9(e)). In addition, the rest of the hydrophobic core residues are still stable until Val26 and Ile30 leave the hydrophobic core (Fig. 9(f)) and global unfolding takes place (Fig. 9(g)). As mentioned, Val26 is crucial for the protein stabilization, and the residue has remained more stable and left the hydrophobic core later than the other residues.

**Figure 10:**
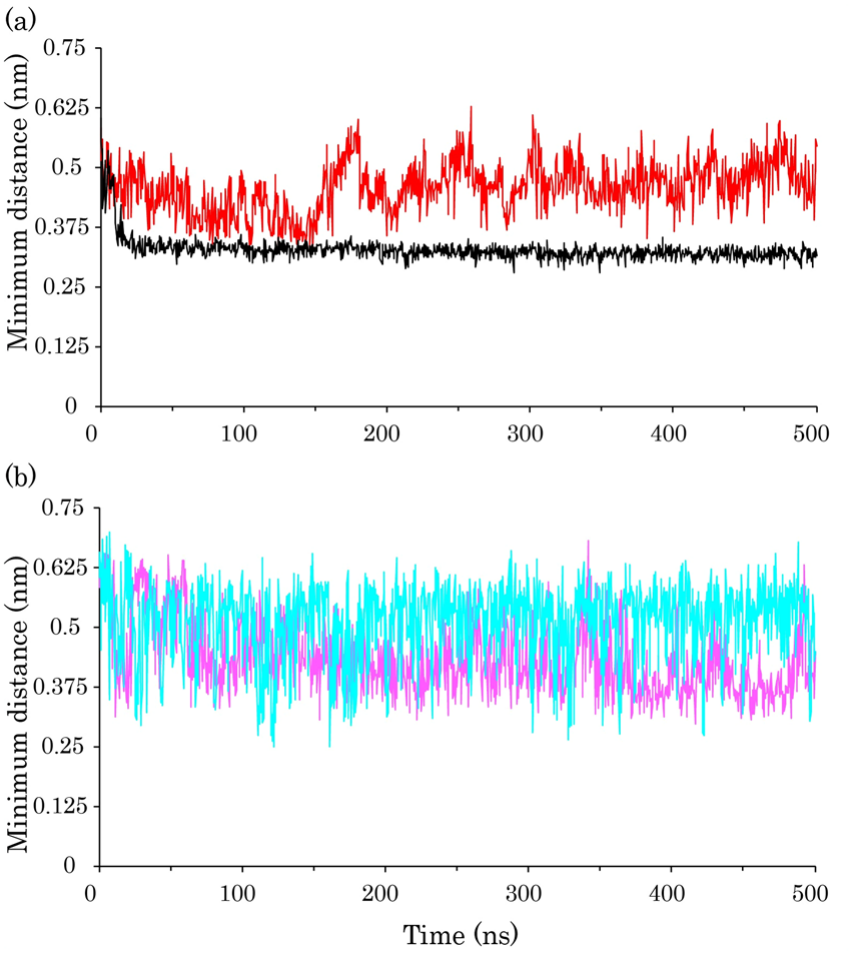
(**a**) The average of minimum distance plot between the hydrophobic core of ubiquitin and SDS tails for the protein in SDS at 300 K (red line) and in SDS at 370 K (black line). The standard deviation of the minimum distance between protein and SDS at 300 K and 370 K was ± 0.5 nm and ± 0.03 nm, respectively. (**b**) The minimum distance plot between the hydrophobic core of ubiquitin and SDS tails for ubiquitin in SDS at 300 and reduced partial atomic charges (magenta line), and in SDS at 370 K and reduced partial atomic charges (cyan line).

To corroborate the insertion of SDS molecules into the hydrophobic core of ubiquitin, we calculated the minimum distance between the SDS tails and the residues of the hydrophobic core (Fig. 10). The results indicated that in the S3, S5, and S6 simulations, at some points during the simulations, the SDS molecules approached the hydrophobic core but they remained at a nearly constant distance away from the hydrophobic core. However, in the S4 simulation, the minimum distance plot reached a plateau below the minimum distance plot of the others. The average minimum distances of SDS molecules were 0.46 ± 0.05 nm, 0.44 ± 0.07 nm, and 0.51 ± 0.08 nm in the S3, S5, and S6 simulations, respectively. This value for the S4 simulation was 0.33 ± 0.27 nm, which indicated that the SDS molecules inserted into the hydrophobic core more favorably than others. Indeed, because of the high temperature at 370 K, the protein begins to partially unfold, and this makes an opportunity for SDS molecules to attack the hydrophobic core and disrupt the internal hydrophobic interactions.

### Binding free energies

To identify which type of the interactions plays the main role in the protein unfolding, we additionally calculated the van der Waals, electrostatic, and nonpolar interactions between the SDS molecules and ubiquitin in the S3, S4, S5, and S6 simulations (Table 3). As demonstrated, both nonpolar and van der Waals interactions were favorable in all simulations, which can be related to the high SDS concentrations (above the CMC). The nonpolar binding free energies (Δ*G*_*npb*_) for the S3, S4, S5, and S6 simulations were −419.2 Kj/mol, −771.2 Kj/mol, −670.7 Kj/mol, and −302.4 Kj/mol, respectively. In all simulations, the hydrophobic interactions predominated because of the high SDS concentrations and saturation of the protein surface by the SDS hydrophobic tails, which is in agreement with previous studies^23,26,63^. These findings suggest that the nonpolar interactions, especially the van der Walls forces, are involved in the interactions of surfactants with ubiquitin at high SDS concentrations. In the S4 simulation, not only were the nonpolar binding energies higher than those of the others, but the electrostatic interactions were also favorable (−10.5 Kj/mol). Furthermore, the total binding energies were higher than the others, indicating that the SDS molecules bind to the protein tightly and lead to global unfolding.

To investigate the binding of SDS molecules to the protein, the total number of contacts between ubiquitin, SDS and water molecules was computed (Table 4). In the S4 simulation, the number of contacts between the protein and water molecules was decreased while it was increased for the protein-SDS complex. These results, as well as the calculated free energies in Table 3, are in good agreement with a previous experimental study on the HSA protein^9^. They have indicated that with increasing the SDS concentrations at temperatures above 80 °C, the strength of contacts between SDS and the protein gradually increased.

**Table 4:** The number of contacts between water molecules and ubiquitin (UBQ-water complex) and the number of contacts between SDS molecules and the protein (UBQ-SDS complex), in S3 and S4 simulations.

System	no. of contacts in UBQ-SDS complex	no. of contacts in UBQ-water complex
S3	305	1464
S4	583	1336

### Effects of SDS on the hydration shell of ubiquitin

The median number of water and SDS molecules in the first hydration shell of the protein was calculated for all simulation systems within R = 0.5 nm^64^ (Table 5). As indicated, the number of water molecules around ubiquitin in the S3 and S4 simulations was remarkably reduced compared to the S1 and S2 simulations because SDS molecules exist in the first hydration shell. The total number of water molecules in the hydration shell (W_N_) of the protein is not constant and this value changes when protein reactions occur^65^. As shown in Table 5, the W_N_ in S3 simulation was more than that of the S4 simulation, suggesting that at a high temperature the water molecules were repelled by SDS. It was confirmed by calculating the number of SDS molecules in the first hydration shell, which in the S4 simulation was significantly more than in the S3 simulation. The total number of hydrogen bonds (H_N_) in the S2 simulation was less than those in the S1 simulation, and this is maybe one of the reasons for the local unfolding in the ubiquitin conformation. The H_N_ value in the S1 simulation was 172, indicating the number of hydrogen bonds which are required for retaining the native conformation of the protein. Our results indicated that the total number of hydrogen bonds made by SDS and water molecules was 170 (146 + 24), providing the H_N_ value which is required in the first hydration shell. Therefore, ubiquitin maintained its native structure in the presence of SDS micelles at 300 K, which was the same as seen in the S1 simulation. In the S4 simulation, the total number of hydrogen bonds made by the SDS and water molecules (129 + 21) was less than that made by only the water molecules in the S2 simulation (166). Possibly, at a high temperature, the SDS and water molecules could not provide the required hydrogen bonds and the first hydration shell around the protein was disrupted and then global unfolding occurred. The hydration shell is determined by the stabilized conformation of proteins, besides the hydrogen bonds, hydrophobic interactions, and van der Waals interactions.

**Table 5:** The number of water molecules and SDS surfactants in the first hydration shell of ubiquitin.

System	no. of SDS	No. of Hbonds^1^	no. of Water	no. of Hbonds^2^
S1	—	—	577	172
S2	—	—	592	166
S3	30	24	419	146
S4	57	21	384	129
S5	30	1	418	152
S6	24	9	425	131

^1^Represents the number of hydrogen bonds between SDS and ubiquitin.

^2^Represents the number of hydrogen bonds between water and ubiquitin.

## Summary

The current study provides information that the surfactant stabilizes the ubiquitin conformation at low temperatures and high SDS concentrations. The Rg and DSSP analyses revealed that ubiquitin loses its native conformation and adopts a random coil structure over the entire simulation time. The results also suggested that the partial atomic charges not only can affect the type and level of interactions in the protein-SDS complex but also can change the orientation, distribution, and assembly of SDS molecules. Moreover, we demonstrated that the SDS surfactant aggregates to form a membrane-like structure and induces global unfolding in the protein conformation at high temperatures. This study demonstrates that maintaining the hydration shell plays an important role in the unfolding mechanism of ubiquitin. The MD simulations also indicated that neither SDS molecules nor temperature can be used alone for inducing the fully unfolded state in the protein structure and both are required. We believe that these findings could be useful in protein biochemistry, protein folding/unfolding and structural biology. Additionally, the SDS surfactant can mimic the biological membrane environment, and investigating its interactions with proteins are of interest in the field of membrane biology. Therefore, our findings can be productive and helpful for any direct examinations of ubiquitin-membrane interactions.

## Electronic supplementary material

Supplementary information
