# Supplementary material for: The Molecular Basis of the Sodium Dodecyl Sulfate Effect on Human Ubiquitin Structure: A Molecular Dynamics Simulation Study
Source: Sci Rep. 2018 Feb 1;8:2150. doi: 10.1038/s41598-018-20669-7 (PMC5794983; doi:10.1038/s41598-018-20669-7)
Supplement: Supplementary file 1 — Supplementary information [file 41598_2018_20669_MOESM1_ESM.doc]

**The Molecular Basis of the Sodium Dodecyl Sulfate Effect on Human Ubiquitin Structure: A Molecular Dynamics Simulation Study**

Majid Jafari^1^, Faramarz Mehrnejad1[[1]](#footnote-2)  , Fereshteh Rahimi^1^, S. Mohsen Asghari^2^

1. Nanobiotechnology Lab, Department of Life Sciences Engineering, Faculty of New Sciences and Technologies, University of Tehran, 14395-1561, Tehran, Iran
2. Department of Biology, Faculty of Sciences, University of Guilan, 4193833697, Rasht, Iran

The supplementary data contain the Dictionary Secondary Structure of Proteins (DSSP), radius of gyration (Rg), and C-alpha root mean square deviation (RMSD) analyses for human ubiquitin in the replicated and extended simulations.

# **DSSP analyses**

As can be seen in Fig.S1, generally, the DSSP of protein in all replicated simulations was the same as each other. However, the native structure of the protein also maintained in the presence and absence of SDS at 300 K. The results also indicated that the local and global unfolding of human ubiquitin occurs in the S3 and S4 simulations, respectively. Fig.S2 demonstrated that the results of DSSP analysis did not significantly change, with extending the simulation time scales. Additionally, the native like secondary structures of human ubiquitin were retained in the presence and absence of SDS at 300 K.


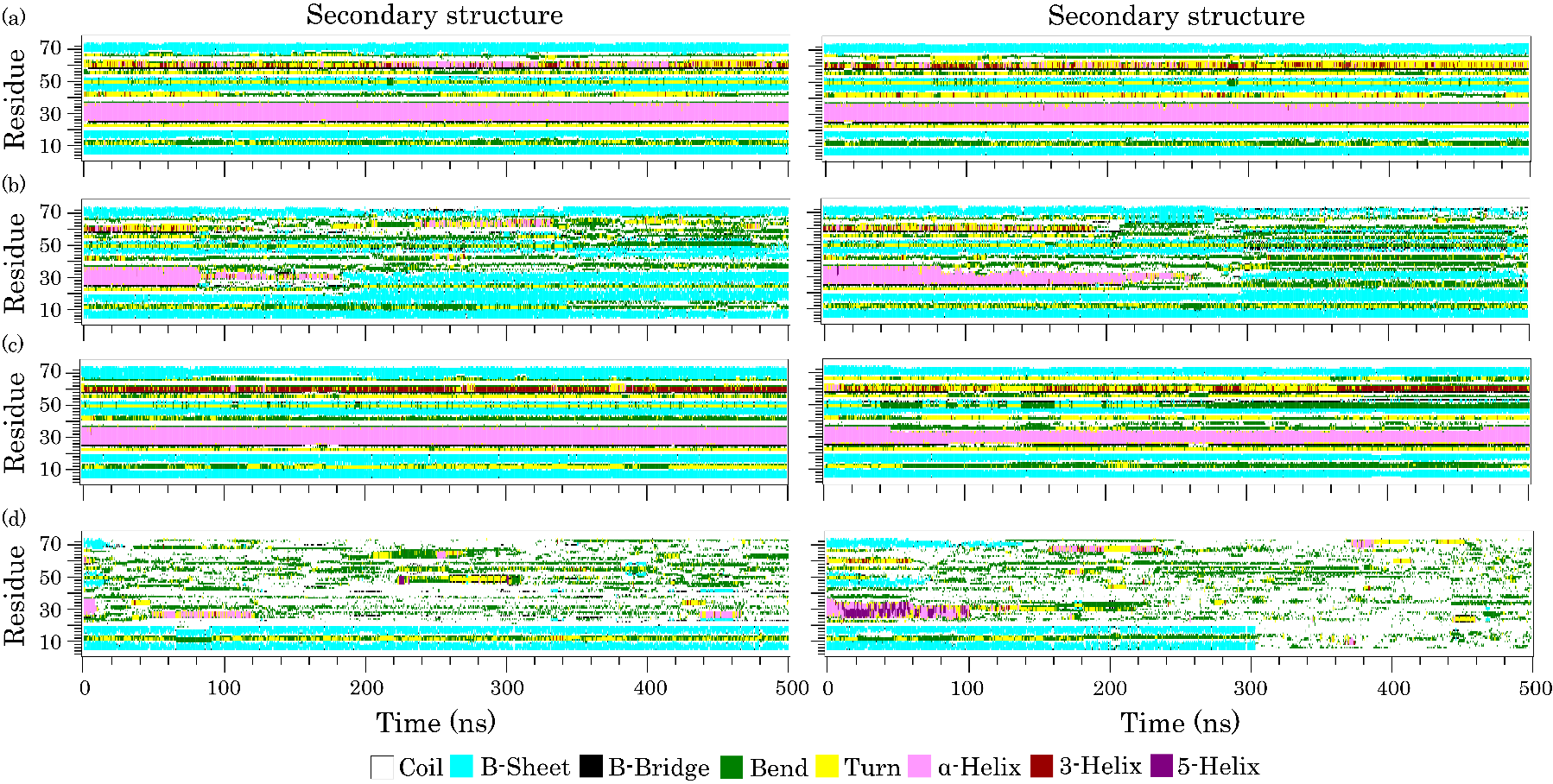


**Figure S1. The Dictionary Secondary Structure of Proteins (DSSP) analysis of human ubiquitin**. Left and right columns represent the second and third simulation replications, respectively. (a), (b), (c), and (d) are S1, S2, S3, and S4 simulation, respectively.


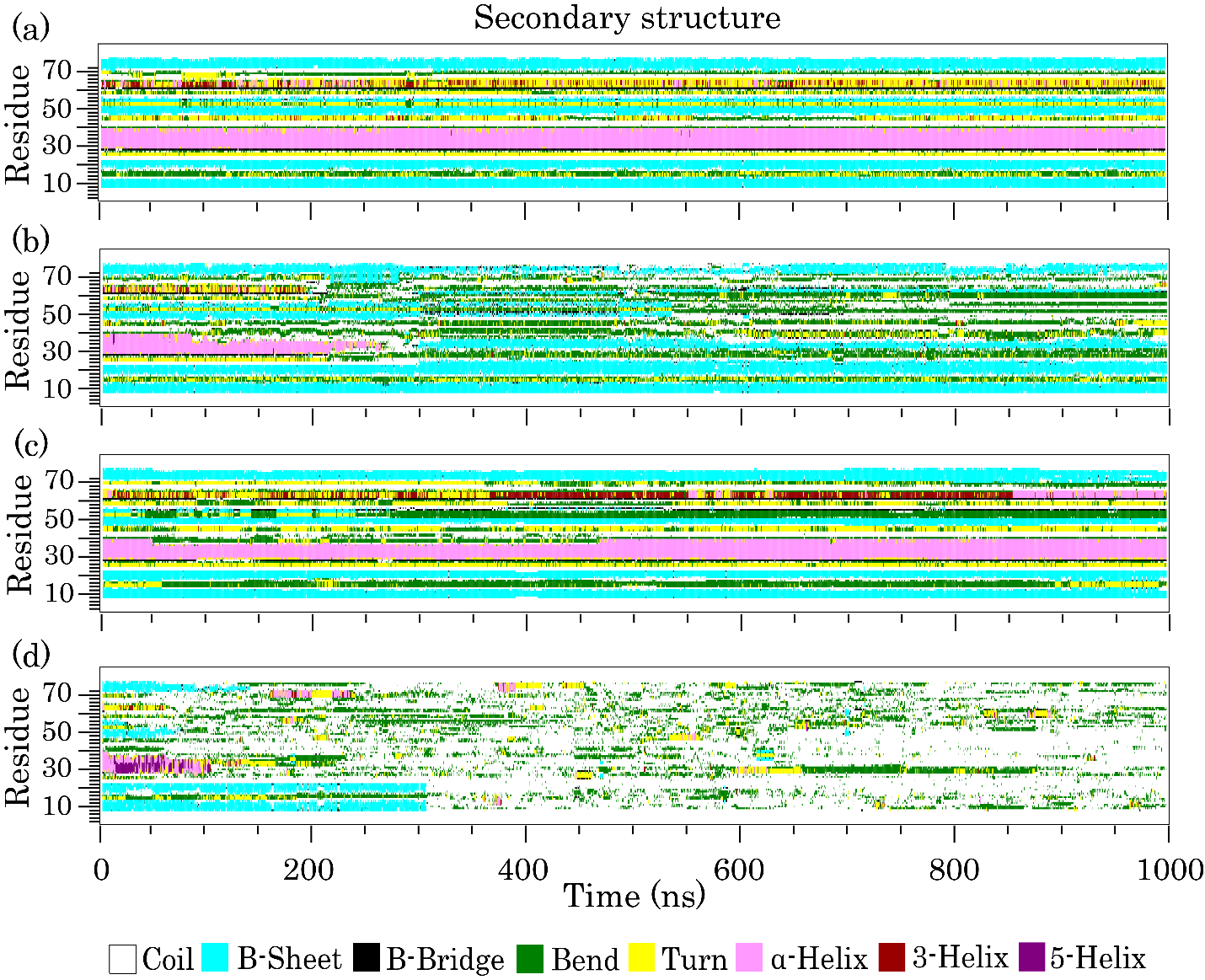


**Figure S2**. **The Dictionary Secondary Structure of Proteins (DSSP) analysis of human ubiquitin.** The secondary structures of human ubiquitin during the simulation time for the extended simulations. (a), (b), (c), and (d) are S1, S2, S3, and S4 simulation, respectively.

# **Rg and RMSD analyses**

During the last 500 ns simulation, the results of Rg and RMSD in S1, S2, and S3 simulations were the same as the first 500 ns, except for S4 simulation. The Rg and RMSD values increased more significantly in the S4 simulation than in the other simulations, with increasing the simulation time. These values drastically increased because of the protein was completely unfolded over the first 500 ns MD simulations.


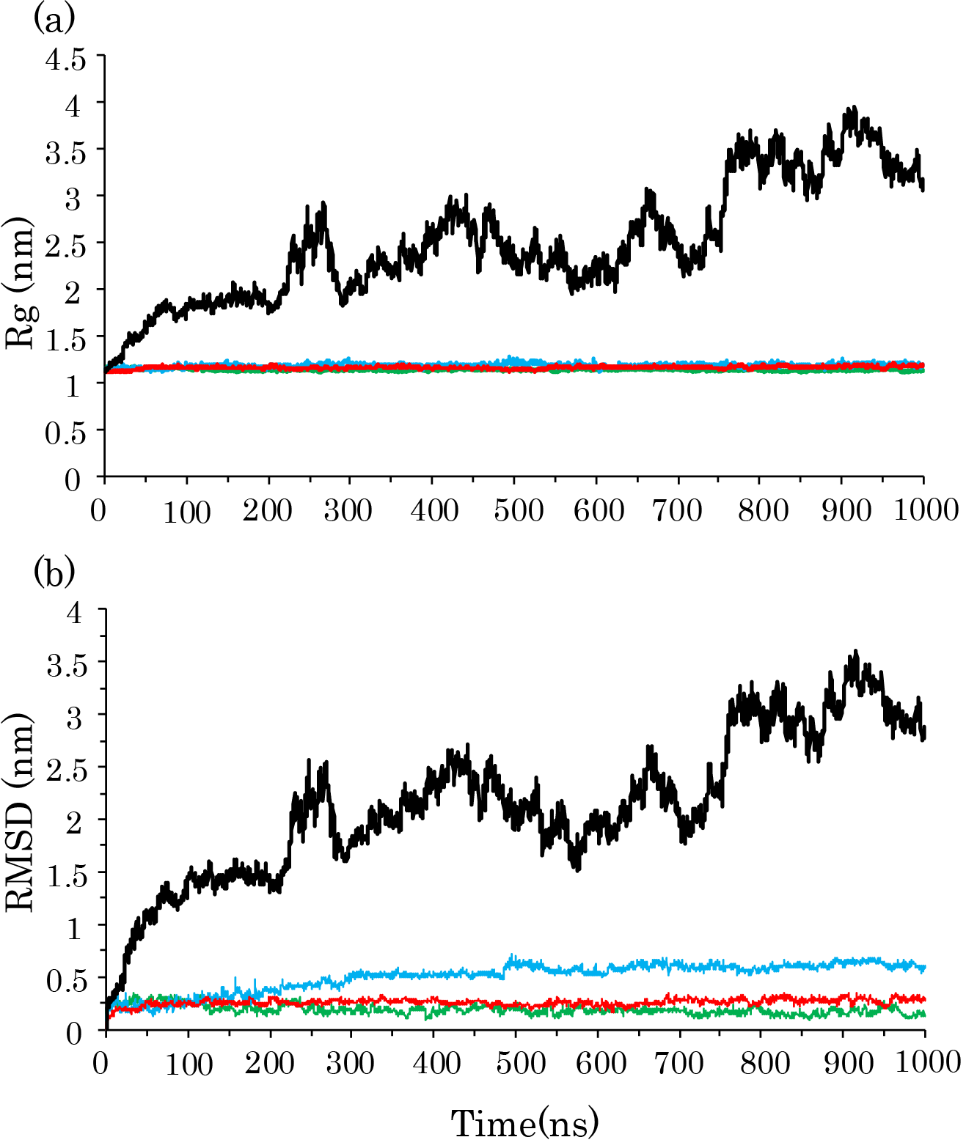


**Figure 3. The radius of gyration (Rg) and C-alpha root mean square deviation (RMSD) of human ubiquitin.** (a) The Rg of human ubiquitin over 1000 ns of simulation. (b) The C-alpha RMSD of the protein backbone during the 1000 ns simulation. In (a) and (b) panels the green, blue, red, and black lines are S1, S2, S3, and S4 simulation systems, respectively.

1. E-mail: [Mehrnejad@ut.ac.ir](mailto:Mehrnejad@ut.ac.ir) [↑](#footnote-ref-2)
